# Resistance gene against *Xanthomonas oryzae pv. Oryzae (Xoo*) in rice: molecular mechanisms and breeding strategies for bacterial leaf blight

**DOI:** 10.3389/fpls.2026.1744367

**Published:** 2026-02-13

**Authors:** Hongrui Jiang, Qina Huang, Changdeng Yang, Yan Liang

**Affiliations:** State Key Laboratory of Rice Biology and Chinese National Center for Rice Improvement, China National Rice Research Institute, Hangzhou, China

**Keywords:** AI-assisted breeding, bacterial, blight, leaf, mechanisms, molecular breeding, oryzae, pathogenic

## Abstract

Bacterial leaf blight (BLB), caused by *Xanthomonas oryzae pv. oryzae* (*Xoo*), is one of the most devastating diseases threatening global rice production. In recent decades, a range of disease resistance genes have been identified in rice. These genes are involved in complex molecular mechanisms, such as the activation of immune receptors and defense signaling pathways, which trigger the plant’s immune response to combat pathogen invasion. Some of these genes have been successfully applied in molecular breeding to develop new disease-resistant varieties. However, traditional breeding methods, which rely heavily on the experience and intuition of breeders, often face limitations in speed and efficiency. With the emergence of artificial intelligence (AI) technologies, there is growing interest in using them to accelerate the breeding of disease-resistant cultivars. This review summarizes the current understanding of the molecular mechanisms underlying BLB resistance, focusing on key resistance genes and their roles in defense responses. It also explores breeding strategies aimed at enhancing resistance and evaluates the opportunities and challenges of AI tools into rice disease resistance breeding.

## 1 Introduction

As a staple crop, rice feeds approximately 50% of the world’s population. With the growth of the global population, ensuring food security has become an urgent priority. BLB, one of the most destructive diseases, poses a major threat to global rice production. The disease is particularly prevalent and severe in Asia, with China, Japan, and India being the most heavily affected (Zhang and Wang, 2013). The spread of BLB is facilitated by multiple pathways, such as contaminated water, wind, insect vectors, and human agricultural activities. The pathogen can rapidly disseminate across rice fields. Climate change—specifically rising temperatures and shifting precipitation patterns—has created favorable conditions for the disease. These climatic shifts have exacerbated the frequency and intensity of BLB epidemics, with pronounced escalation observed in tropical and subtropical agroecosystems (Niones et al., 2022).

To combat pathogen infection, plants have evolved a two-layer defense system. The first layer of the immune system involves pattern recognition receptors (PRRs) on plant cell surfaces that detect conserved pathogen-associated molecular patterns (PAMPs), such as flagellin and chitin, upon pathogen attack. This recognition triggers a range of responses, including cell wall modifications, callose deposition, and activation of defense-related proteins, collectively known as PAMP-triggered immunity (PTI) (Jones and Dangl, 2006). In the coevolution of host-microbe interactions, pathogens have evolved mechanisms to deliver effector proteins into plant cells, thereby suppressing PTI responses. The second layer of immunity, effector-triggered immunity (ETI), is activated by resistance proteins that specifically recognize pathogen effectors and initiate a robust defense response (Jones and Dangl, 2006). This highly specialized defense mechanism triggers a cascade of responses at the infection site, including shifts in ion concentrations (e.g., Ca²^+^, K^+^, H^+^), the accumulation of superoxide and nitric oxide (Nürnberger et al., 2004). To restrict pathogen invasion, infected plant tissues often initiate a hypersensitive response (HR), manifesting as rapid, localized cell death (Ngou et al., 2022).

Advances in understanding the rice-*Xoo* interaction have identified key disease-resistance genes. These findings provide crucial targets for breeding programs by elucidating the rice immune system and its genetic basis (Wang et al., 2013; Chen et al., 2021; Xu et al., 2022). Breeding practices have shown that developing resistant rice varieties is the most cost-effective, efficient, and environmentally sustainable method for controlling BLB. The application of Marker-Assisted Selection (MAS) has enabled the successful breeding of new high-yielding and disease-resistant rice varieties (Islam et al., 2024; Dong et al., 2025). For complex trait integration, MAS focuses on a limited number of genes, while data-driven AI can simultaneously optimize multiple traits, such as yield, quality, and disease resistance. MAS precisely targets known resistance genes but is constrained by existing knowledge, whereas AI predicts complex genetic patterns, offering greater flexibility despite challenges in data and modeling (Chukwu et al., 2019; Kim et al., 2019a; Oliva et al., 2019; Gupta et al., 2023). This review summarizes the molecular mechanisms of BLB resistance in rice and further discusses the application of MAS and AI-assisted breeding, providing a roadmap for the development of resistant varieties.

## 2 The infection process and pathogenic mechanisms of *Xoo*

*Xoo* primarily infects rice leaf tissues through the stomata, which are concentrated at the leaf tips and edges. Upon entry, *Xoo* invades the vascular tissues, proliferating in the intercellular spaces, and spread throughout the leaf via the vascular bundles. Extensive proliferation of *Xoo* in vascular tissues causes vessel occlusion (NiÑO-Liu et al., 2006). In the early stages of infection, rice leaves develop yellow streaks at the tips and edges, which gradually expand across the leaf surface. As the infection progresses, the infected leaves turn grayish white, eventually drying out and dying (Naqvi, 2019).

During infection, *Xoo* must overcome multiple immune barriers in rice, including the waxy cuticle, the suberin layer, and the cell walls between plant cells, to access the vascular tissues. *Xoo* produces several adhesin-like proteins that facilitate its attachment to the leaf surface. The absence of *XadA* (*Xanthomonas* adhesin-like protein A) significantly reduces the pathogenicity of *Xoo* on the leaf surface, and a similar decrease in virulence is observed in the absence of *XadB* (*Xanthomonas adhesin-like protein B*). Notably, mutations in the *XadA* and *XadB* genes do not impact the virulence of *Xoo* when inoculated onto wounded rice tissue (Das et al., 2009). *Xoo* secretes various extracellular enzymes to degrade the host’s tissue barriers. These enzymes primarily include pectinases, proteases, and cellulases. The *Xoo* genome encodes at least four homologous enzymes involved in the degradation of homogalacturonan (HG), a key component of pectin: polygalacturonase (*pglA*), pectin methyl esterase (*pmt*), and two pectate lyases (*peI* and *peII*). Among these, *pglA* is the major pectinase produced by *Xoo*. However, compared to cellulases and xylanases, HG-degrading enzymes likely do not play a central role in *Xoo*’s pathogenicity (Wilson et al., 2016). XoCP, a cysteine protease secreted by *Xoo*, is significantly upregulated upon contact with rice leaves. Knockout of *XoCP* reduces virulence by impairing tissue invasion (Wang et al., 2017). Additionally, *Xoo* secretes xylanase (XynB), a key component of cellulases. Mutation of the *xynB* gene impairs xylanase secretion, which in turn affects the virulence of *Xoo* (Rajeshwari et al., 2005). Several other secreted factors, including extracellular polysaccharides (EPS) (Yun et al., 2006), lipopolysaccharides (LPS) (Girija et al., 2017), quorum sensing networks (Zhou et al., 2017) and cyclic-di-GMP signaling (Yang et al., 2019), also contribute to the pathogenicity of *Xoo* during rice infection.

Effector proteins are key virulence factors that promote infection by disrupting plant immune signaling. The injection of these effector proteins into rice cells by *Xoo* is mediated by a conserved Type III secretion system (T3SS), which is essential for bacterial infection (Alfano and Collmer, 2004; Ryan et al., 2011). T3SS of *Xoo*, encoded by the *hrp* (*h*ypersensitive *r*esponse and *p*athogenicity) genes and disruption of this system severely abolishes pathogenicity (Büttner and Bonas, 2006). Transcription activator-like effectors (TALEs) represent a major family of effector proteins (Ji et al., 2014). Through binding to host gene promoters, these effectors activate susceptibility genes expression, including *OsSWEET11* (Yuan et al., 2010), *OsSWEET13* (Zhou et al., 2015), *OsSWEET14* (Streubel et al., 2013) and *OsTFX1* (Sugio et al., 2007), thereby enhancing host susceptibility and facilitating BLB development (Sun et al., 2021). Thus, the pathogenicity of *Xoo* is influenced by multiple processes, involving the coordinated action of adhesion factors, cell wall-degrading enzymes, and a range of effector proteins delivered via the T3SS to suppress host immunity.

The targets and molecular mechanism of most non-TALE effectors in rice remain largely unknown, only few non-TALE genes have been characterized. OsVOZ2 interact with non-TALE XopN induce calcium deposition and hydrogen peroxide accumulation against *xoo* (Chakravortty et al., 2013). XopR of *xoo* interact with OsBIK1, suppressing PAMP-triggered stomatal closure in *Arabidopsis* (Wang et al., 2016). OsRLK135, OsBAK1 and OsSERK1 can also interact with non-TALEs to initiate immune response (Yamaguchi et al., 2013, Yamaguchi et al., 2014; Wang et al., 2016). Furthermore, non-TALEs interact with OsPUB44 and OPR1C to inhibit rice resistance to *xoo* (Ishikawa et al., 2014; Ji et al., 2022). The dual functionality of non-TALEs—their ability to either trigger or suppress defenses—highlights a sophisticated regulatory network that fine-tunes rice immunity against *Xoo*.

## 3 Major BLB resistance genes in rice

**Figure 1:**
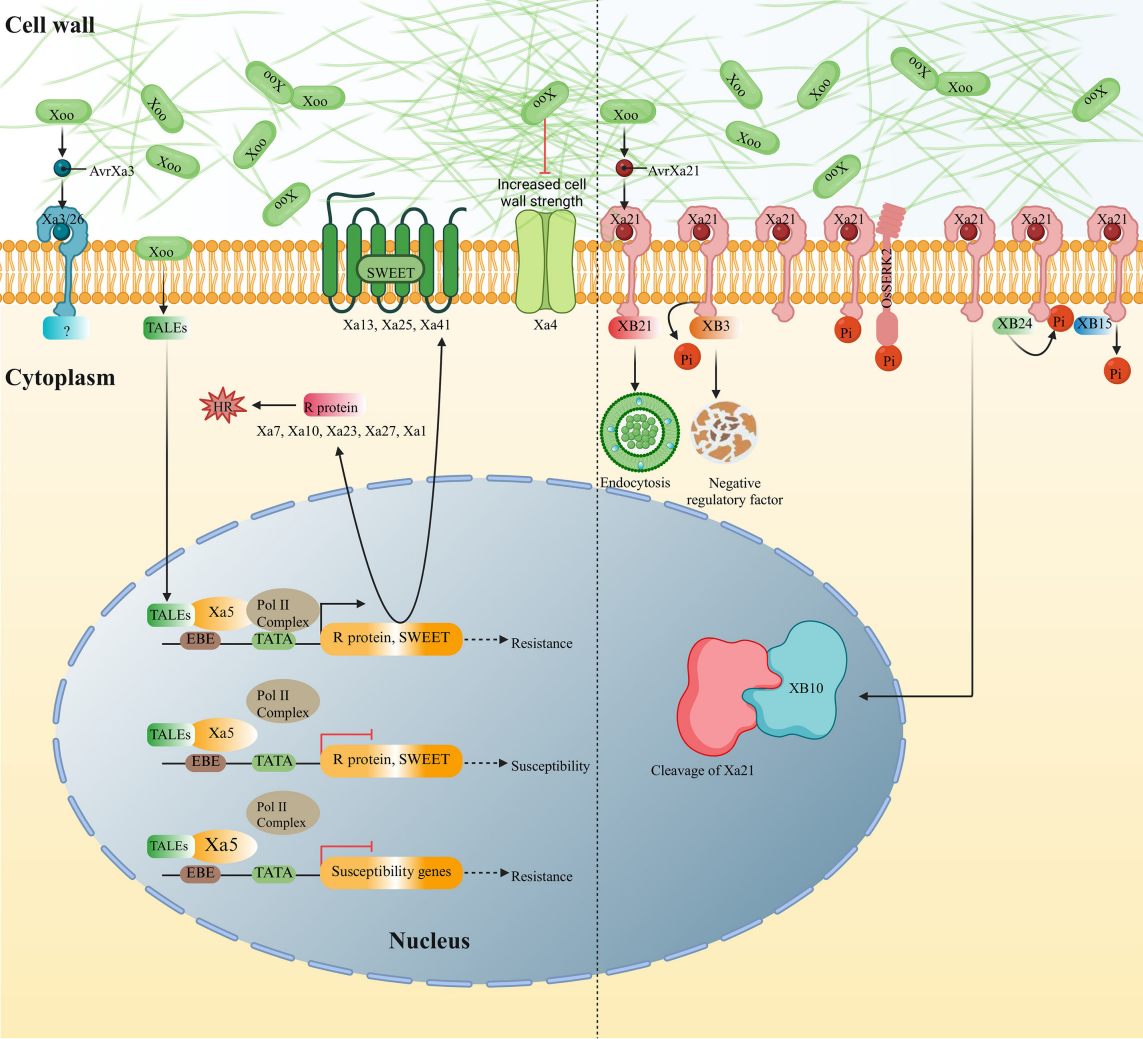
Molecular mechanism of the cloned gene conferring resistance to BLB. (left) This schematic diagram illustrates the key molecular processes involved in the resistance conferred by the cloned resistance gene. Upon pathogen recognition, Xa21 and Xa3/Xa26 recognize AvrXa21 and AvrXa3 to activate downstream signaling pathways, respectively. The TALEs proteins are secreted by Xoo entering the plant cell and specifically bind to the promoter regions of susceptible rice genes and activate their expression (SWEET), thereby facilitating infection. The xa5 gene confers resistance by recognizing and counteracting the activity of the TALEs proteins, blocking their ability to induce susceptible gene expression. The R protein is activated by TALE proteins, the activation of R proteins initiates a hypersensitive response (HR), characterized by rapid cell death. This local cell death limits the spread of the pathogen and acts as an effective defense mechanism. The Xa4 protein promotes cellulose synthesis to resist invasion by the BLB. The immune signaling pathways mediated by *Xa21*, triggered by *Xoo* (right). Several XA21 binding proteins, including XB3, XB10, XB15, XB21, XB24, OsSERK2 are involved in regulating XA21-mediated resistance. XB3 is essential for the accumulation of XA21. Upon activation, XA21 undergoes phosphorylation by XB3, which triggers the release of negative regulatory factors, promoting disease resistance. XB10 interacts with the cleaved form of XA21, which translocates its intracellular kinase domain to the nucleus and activates the immune response. XB15 dephosphorylates the auto-phosphorylated XA21, thereby dampening XA21-mediated resistance. XB21, an auxilin-like protein, is predicted to function in clathrin-mediated endocytosis, modulating XA21-mediated immunity. XB24 interacts with XA21 and promotes its autophosphorylation, thereby maintaining XA21 in an inactive state. OsSERK2 is a regulator involved in multiple receptor kinase-mediated immune signaling pathways, capable of forming a complex with XA21 and undergoing reciprocal phosphorylation.

Genetic resistance in host plants provides an economical, effective, and environmentally sustainable approach to controlling plant diseases and reducing yield losses. To date, at least 48 BLB resistance genes have been identified in rice, including those derived from wild rice species. Among these, 15 resistance genes (*xa5*, *xa8*, *xa13*, *xa15*, *xa19*, *xa20*, *xa24*, *xa25*, *xa28*, *xa31*, *xa32*, *xa34*, *xa41*, *xa42* and *xa44*) are recessive, while the remaining are dominant. At least 12 BLB resistance genes have been successfully cloned, including *Xa1*, *Xa3/Xa26*, Xa4, *xa5*, *Xa7*, *Xa10*, *xa13*, *Xa21*, *Xa23*, *xa25*, *Xa27* and *xa41* (**Table 1**). These cloned resistance genes can be classified into five categories based on ​​their involvement with​​ different molecular modules: LRR (leucine-rich repeat) receptor-like kinases: *Xa21*, *Xa3/Xa26*, basic transcription factors: *Xa5*, R proteins: *Xa7*, *Xa23*, *Xa27*, sucrose transporters: *xa13*, *xa25*, *xa41*, and ​​others: *Xa1*, *Xa4*​​ (**Figure 1**).

**Table 1:** Summary of cloned gene resistance to BLB.

Gene	Categories	Encode protein	Gene function	Cognate Avr genes	Encode protein	Resistance to *Xoo* strain	References
*Xa21*	LRR Receptor Kinases	LRR-RLK	pathogen recognition and signaling processing	*RaxX*	Unknown	Philippine and Japanese Races	(SONG et al., 1995; Chen et al., 2010a; Park and Ronald, 2012)
*Xa3/Xa26*	LRR Receptor Kinases	LRR-RLK	pathogen recognition and signaling processing	*AvrXa3*	TAL effector	Chinese, Philippine, and Japanese races	(Sun et al., 2004; Xiang et al., 2006; Cao et al., 2007)
*xa5*	Basic transcription factor	TFIIA transcription factor	Inhibition of TALE protein-mediated transcription of susceptibility genes	*Avrxa5* *PthXo7*	TAL effector	Philippine races I, II, III	(Jiang et al., 2006; Gu et al., 2009; Yuan et al., 2016)
*Xa7*	R proteins	Executor R protein	TALE protein-mediated regulation of immune response activation	*AvrXa7*	TAL effector	Philippine races	(Yang et al., 2000; Chen et al., 2021)
*Xa10*	R proteins	Executor R protein	TALE protein-mediated regulation of immune response activation	*AvrXa10*	TAL effecto	Philippine and Japanese Races	(Gu et al., 2007; Tian et al., 2014)
*Xa23*	R proteins	Executor R protein	TALE protein-mediated regulation of immune response activation	*AvrXa23*	TAL effector	Indonesian races	(Wang et al., 2013, 2015; Xu et al., 2022)
*Xa27*	R proteins	Executor R protein	TALE protein-mediated regulation of immune response activation	*AvrXa27*	TAL effector	Chinese strains and Philippine race 2 to 6	(Gu et al., 2005, 2009)
*xa13*	Sucrose transporters	SWEET-type protein	Variation in the promoter region, not regulated by TALE proteins	*PthXo1*	TAL effector	Philippine race 6	(Yuan et al., 2010, 2011; Gao et al., 2018; Kim et al., 2019b)
*xa25*	Sucrose transporters	SWEET-type protein	Variation in the promoter region, not regulated by TALE proteins	*PthXo2*	TAL effector	Chinese races Zhe 173 and Philippine races 9	(Liu et al., 2011; Zhou et al., 2015; Sundaram et al., 2018)
*xa41*	Sucrose transporters	SWEET-type protein	Variation in the promoter region, not regulated by TALE proteins	*AvrXa7/PthXo3/TalC*	TAL effector	Various *Xoo* strains	(Hutin et al., 2015; Kim et al., 2020)

### 3.1 The signaling pathway mediated by BLB resistance proteins

The rice resistance genes *Xa3/Xa26*, *xa5*, *Xa7*, *Xa21*, *Xa23*, *Xa27* confer resistance to numerous strains of rice bacterial blight. The C-terminal kinase domain of *Xa3/Xa26* is essential for its resistance to BLB. Notably, pathogen infection does not induce the expression of *Xa3/Xa26* (Cao et al., 2007). *OsTPI1.1* encodes a triosephosphate isomerase (TPI) that catalyzes the reversible conversion of dihydroxyacetone phosphate to glyceraldehyde-3-phosphate. Downregulation of *OsTPI1.1* significantly impairs XA3/XA26-mediated resistance. The defense response involves OsTPI1.1 through its TPI activity, which is notably enhanced upon interaction with XA3/XA26 (Liu et al., 2018).

*Xa5* encodes the γ subunit of the general transcription factor TFIIA (Høiby et al., 2007). A mutation at position 39, where valine is substituted by glutamic acid (resulting in a susceptible protein), impairs the function of *Xa5* (Jiang et al., 2006). This mutation may confer resistance to *Xoo* by suppressing bacterial transmission without affecting its proliferation (Lyer-Pascuzzi et al., 2008). The recessive xa5 allele encodes a TFIIAγ variant that prevents *TALE* binding, thereby blocking *SWEET11* transcriptional activation. TALE-induced expression of susceptibility genes (e.g., *Xa13* and *Xa41*) is prevented in *xa5* plants or *TFIIAγ5-RNAi* lines, conferring enhanced resistance to BLB. In the absence of *TFIIAγ5*, another *TFIIAγ* gene, *OsTFIIAγ1*, can be activated by PthXo7, explaining why PthXo7-containing *Xoo* strains overcome *xa5*-mediated resistance (Ma et al., 2018). Additionally, TFIIAγ5 is required for TALE-mediated transcriptional activation of key defense-related genes such as *Xa27* and *Xa23*, which are crucial for plant immunity against this disease (Yuan et al., 2016).

The *Xa7* gene encodes a protein of unknown function, composed of 113 amino acid residues. Its promoter contains an effector-binding element (EBE) recognized by the AvrXa7 and PthXo3 effectors. Constitutive expression of *Xa7* leads to continuous activation of immune responses but also inhibits normal rice growth (Chen et al., 2021).

The molecular mechanisms of Xa21-mediated immunity are extensively characterized. The pathogen-derived RaxX protein, a tyrosine-sulfated secretory molecule exported through the type I secretion system of *Xoo*, has been identified as the cognate ligand triggering XA21-mediated immune activation. This ligand-receptor interaction establishes the molecular basis for pathogen recognition in the XA21 signaling pathway (Pruitt et al., 2015). The tyrosine-sulfated RaxX (AvrXa21) demonstrates high-affinity binding to XA21 (Luu et al., 2019). *OsSERK2* functions as a regulatory component in multiple receptor kinase-mediated immune signaling pathways, including those governed by *XA21*, *XA3*, and *OsFLS2*. XA21-OsSERK2 complex mutually phosphorylates to regulate rice immunity. OsSERK2-knockdown rice lines exhibit compromised XA21 and XA3/XA26-mediated immunity against *Xoo* (Chen et al., 2014).

Several *Xa21*-interacting proteins (XB) have been identified. *XB3*, which encodes an E3 ubiquitin ligase, is crucial for maintaining Xa21 protein stability. Upon pathogen infection, Xa21 phosphorylates XB3, leading to the ubiquitination and degradation of negative regulators involved in the immune response, thereby activating the disease resistance signaling pathway (Wang et al., 2006). XA21 interacts with a WRKY transcription factor, OsWRKY62 (XB10), which encodes two splice variants (OsWRKY62.1 and OsWRKY62.2). These variants are partially localized in the nucleus and act as negative regulators of rice resistance. They are involved in the modulation of both basal immunity and race-specific immune responses (Peng et al., 2008). *XB15* encodes the PP2C protein phosphatase, which dephosphorylates the auto-phosphorylated Xa21, thus negatively regulating the immune response mediated by Xa21 (Park et al., 2008). The auxin-like protein XB21 also interacts with XA21. Overexpression of *XB21* enhances resistance to *Xoo*, potentially through a mechanism involving endocytosis mediated by clathrin (Park et al., 2017). *XB24* encodes an ATPase that interacts with Xa21 through its ATPase domain. This interaction promotes the phosphorylation of Xa21 while simultaneously inhibiting the immune response mediated by Xa21 (Chen et al., 2010b). In the absence of infection, the ATPase XB24 constitutively interacts with the regulatory juxtamembrane domain of XA21, facilitating phosphorylation of conserved serine/threonine motifs that stabilize the inactive formation. Pathogen recognition triggers dissociation of the XA21-XB24 complex, activating XA21 kinase activity and initiating phosphorylation-dependent defense signaling cascades (Chen et al., 2010b).

*Xa23* shares the same open reading frame (ORF) as the susceptible allele *xa23*, with a polymorphism of 7-bp in the promoter region. This difference allows *Xa23* to be induced by the *AvrXa23* effector, while the *xa23* allele remains unresponsive. Upon expression, *Xa23* triggers a hypersensitive response (HR), leading to cell death, thereby limiting pathogen proliferation and inhibiting further *Xoo* invasion (Wang et al., 2015). *AvrXa23* is widely present in 38 prevalent *Xoo* strains, which likely contributes to the resistance conferred by *Xa23* (Wang et al., 2013).

The *Xa27* gene, derived from tetraploid *Oryza rufipogon*, confers resistance to *Xoo* strains from various countries (Gu et al., 2005). The BLB AvrXa27 effector binds to the promoter of the *Xa27* gene and induces its expression. The Xa27 protein is then secreted into the apoplast, leading to inhibition of bacterial growth. The correct localization of Xa27 is required for resistance to BLB. *Xa27* and the susceptible allele *xa27* share an identical coding sequence, with only two differences in the promoter region (Wu et al., 2008). Additionally, the disease resistance mediated by *Xa27* relies on the function of the basic transcription factor *Xa5* (Gu et al., 2009).

### 3.2 Dual recognition strategies in rice resistance to BLB: direct R-Avr interactions and effector-mediated promoter binding

The interactions between rice resistance proteins and BLB avirulence proteins adheres to the “gene-for-gene” theory.In rice, various genes have been found to correspond to BLB avirulence proteins: *Xa1*, *Xa10*, *xa13*, *xa25*, *xa41*.

The *Xa1* encodes a protein of 1,802-amino acid, containing a nucleotide-binding domain (NBS) and a leucine-rich repeat (LRR) region (Belkhadir et al., 2004). Both mechanical injury and pathogen infection induce the expression of *Xa1* (Yoshimura et al., 1996). Xa1 recognizes multiple TALEs secreted by *Xoo*, including PthXo1, Tal4, and Tal9d, leading to the activation of downstream gene expression and conferring resistance to the pathogen in rice. A truncated interfering TALE (iTALE) that lacks the transcriptional activation domain is capable of disrupting the resistance conferred by Xa1 (Ji et al., 2016).

The *Xa10* gene encodes a small protein, consisting of 126 amino acid residues, conferring resistance to certain Philippine races of *Xoo*, was first identified from rice cultivar Cas 209 (Lee et al., 2003; Gu et al., 2007). The *Xa10* promoter region includes an effector-binding element (EBE) recognized by the *AvrXa10* effector, which initiates *Xa10* expression. *Xa10* specifically recognizes the *AvrXa10* protein from the pathogen, disrupts intracellular Ca²^+^ homeostasis, triggers programmed cell death and restricts further pathogen invasion (Tian et al., 2014).

The *xa13*, *xa25*, and *xa41* genes are recessive resistance genes against BLB, encoding the Sugar Will Eventually be Exported Transporter (SWEET) proteins. These SWEET proteins are involved in the process of sugar efflux from cells, a mechanism that plays a crucial role in various biological processes, including nectar secretion and the transport of sugars to progeny tissues for their development (Chen, 2013). In rice, 22 genes belong to the *SWEET* gene family (Chen et al., 2010a). A key difference of 18 base pairs in the promoter region prevents the hijacking of *xa13* by the TAL effectors PthXo1 secreted by *Xoo*, resulting in the expression of a resistant phenotype (Chu et al., 2006). *Xa13* facilitates pathogen-induced copper redistribution in rice by mediating interactions between OsCOPT1 and OsCOPT5. This interaction reduces xylem Cu^2+^ concentration, alleviating copper toxicity to *Xoo* and promoting its proliferation (Yuan et al., 2010). The promoter region of *Xa25* contains sequences that can be recognized and activated by PthXo2-like TALE proteins secreted by *Xoo*. When susceptible rice plants are infected with *Xoo* strains expressing PthXo2, the PthXo2 protein activates the expression of Xa25, facilitating the transport of sucrose into the apoplast, thereby supporting bacterial infection of rice tissues. Conversely, the promoter of the resistant *xa25* allele differs from that of the susceptible *Xa25* allele, lacking the PthXo2 EBE and thereby conferring resistance (Liu et al., 2011). The *xa41* gene (*Os11N3*, *OsSWEET14*) encodes a sucrose transporter protein of 303 amino acids. Similar to *xa13* and *xa25*, it can be induced by TALE proteins secreted by various *Xoo* strains. The promoter region of *xa41* exhibits an 18-base pair deletion, which prevents activation of Xa41 protein expression by TALE effectors (Hutin et al., 2015).

### 3.3 Other disease-resistance genes

The *Xa4* gene encodes a cell wall-associated kinase that promotes cellulose synthesis and inhibits cell wall loosening, thereby increasing cell wall strength. The *Xa4* gene confers resistance to BLB by reinforcing the cell wall, which also enhances stem mechanical strength. *Xa4* confers pleiotropic effects on important agronomic traits, increasing grains per panicle while reducing panicle number per plant and 1000-grains weight. Consequently, plant height is reduced, which enhances lodging resistance without compromising yield (Hu et al., 2017). The widespread use of *Xa4* can be attributed to its association with multiple beneficial agronomic traits.

## 4 Molecular breeding strategies for enhancing BLB resistance in rice

Pyramiding of resistance genes, gene editing are two major molecular breeding strategies that utilize resistance genes. However, food safety concerns and regulatory restrictions in some countries have significantly hindered the adoption of susceptibility gene editing and engineered disease resistance proteins. The pyramid of resistance genes through MAS strategies represents an effective way to attain durable resistance, as it is free from political and social issues and is widely adopted by breeders. *xa5, Xa7, xa13, Xa21*, and *Xa23*, which confer resistance to various xoo strains, have been widely applied in rice breeding programs (Latif et al., 2024).

### 4.1 Pyramiding of resistance genes

Ullah et al. introduce four BLB resistance genes *Xa4*, *xa5*, *xa13*, and *Xa21* into the indica rice variety Basmati-385, an indica rice cultivar with much sought-after qualitative and quantitative grain traits. The pyramided lines with quadruplet or triplet R genes showed the highest level of resistance compared to doublet or singlet R genes. Further, The pyramided lines with quadruplet and triplet R genes (*Xa4*, *xa5*, *xa13*) exhibited high level resistance to BLB, without any yield penalty (Ullah et al., 2022). BLB resistance genes are often pyramided with blast resistance genes to achieve durable resistance. MAS successfully pyramided the blast resistance gene Pi9 and the BLB resistance gene *Xa23* in the thermosensitive male-sterile line GZ63S and its hybrid progeny Liangyou6326, conferring high resistance to all eight tested *Xoo* strains and broad-spectrum resistance to both leaf and neck blast (Ni et al., 2015). Pyramiding three bacterial leaf blight resistance genes (*xa5, xa13, Xa21*) and two blast resistance genes (*Pi9, Pb1*) into the rice variety BRRI dhan63 resulted in lines with diverse gene combinations. All pyramided lines showed significantly enhanced resistance to BLB or blast compared to the original cultivar (Nihad et al., 2023). MAS has been successfully applied in the development of multiple rice varieties with enhanced disease resistance and provides a reliable method for obtaining highly effective BLB-resistant rice lines.

### 4.2 Creating disease-resistant cultivars using gene editing technology

Emerging genome-editing technologies, including TALENs, CRISPR/Cas9, and prime editing (PE), allow precise modification of crop genomes. These technologies have been successfully applied in model species and hold great potential for enhancing plant disease resistance. Gene editing confers disease resistance in rice primarily through two strategies: modifying susceptibility genes or engineering resistance proteins based on structure. TALENs represent one of the earliest engineered nuclease-based tools. TALEN-based technique was employed to knockout the *Xa5* gene, resulting in increased BLB resistance in the edited rice lines. Different knockout variants exhibited varying levels of resistance, suggesting that amino acid substitutions at different positions within the *Xa5* gene can modulate the rice’s resistance to BLB (Han et al., 2020). But the design and construction of TALENs are labor-intensive and technically demanding, requiring the synthesis of custom-designed DNA-binding domains for each target sequence. These high costs and time demands make widespread adoption difficult.

Beyond directly editing susceptibility genes, enhancing disease resistance in rice can be achieved through knock-in or knock-out EBE element in resistance genes and susceptibility genes respectively. CRISPR/Cas9 has emerged as the most widely adopted genome-editing technology because of its simplicity, high efficiency, and ease of use. A 149-bp sequence containing a 31-bp pathogen-responsive element was edited in the promoter of the sugar transporter gene *Xa13* using the CRISPR/Cas9 system. The edited rice lines exhibited resistance to *Xoo* infection due to the failure to activate susceptibility genes, without compromising key agronomic traits or fertility (Li et al., 2019a). Off-target effects, where Cas9 cleaves unintended sites, remain a primary concern, especially for targets with high sequence similarity. Despite the development of high-fidelity Cas9 variants to mitigate this, the risk is still non-negligible. Prime editing (PE) is a precision genome-editing technology that represents a significant advance over CRISPR/Cas9. It employs a nCas9-reverse transcriptase fusion and a pegRNA to execute targeted nucleotide substitutions, insertions, and deletions with high accuracy. PE was employed to knock-in the EBE-binding element of the *Xa41* into the promoter of dysfunctional *Xa23*, enabling *Xoo*-induced, TALE-dependent BLB resistance (Gupta et al., 2023). Prime editing (PE) remains an emerging technology and still requires comprehensive validation across a wide range of experimental models and applications. In conclusion, gene pyramiding is a widely adopted strategy for durable resistance, while gene editing offers a precise alternative.

## 5 ​​Next-generation breeding: an integrated AI framework for developing disease-resistant cultivars

Molecular breeding strategies, including MAS and gene editing, have significantly advanced crop improvement. Their application is frequently restricted by a reliance on cloned major defense-related genes, limiting their ability to tackle complex traits. To overcome these limitations, AI is being increasingly integrated practices through technologies like genomic selection (GS) and phenomic selection (PS). The use of AI in developing BLB-resistant cultivars is still in the early stages. Nevertheless, it has the potential to enhance breeding efficiency and pave the way for innovative disease-resistance strategies.

AI algorithms encompass machine learning (ML), deep learning (DL), and large-scale models. ML techniques have proven highly effective for analyzing complex plant-omics datasets and addressing the non-linear relationships between genotypes and phenotypes, such as through Deep Neural Network-based Genomic Prediction (DNNGP), which leverages multi-omics data for genomic predictions in plants (Liu et al., 2024). DL, a subfield of ML, utilizes artificial neural networks to automatically extract relevant features from data, making it particularly adept at handling high-dimensional, unstructured datasets. Convolutional Neural Networks (CNNs) are a cornerstone of deep learning, largely due to their outstanding performance in processing complex, large-scale, and high-resolution image data (Li et al., 2019b). Large models are deep learning architectures characterized by a vast number of parameters and training on extensive datasets. These systems demonstrate strong capabilities in addressing complex challenges, with validated success across multiple domains (Wang et al., 2023).

### 5.1 AI-assisted GS for enhanced disease resistance

ML and DL facilitate scalable, objective screening with reproducible and quantitative outputs (Merrick et al., 2022). The Support Vector Machine (SVM) regression model achieved prediction accuracy comparable to or exceeding that of traditional parametric models (Merrick et al., 2022). A recent study utilized kinship matrices integrated into three machine learning frameworks—Random Forest (RF), Support Vector Machine (SVM), and Light Gradient Boosting Machine (LightGBM)—to predict resistance to multiple diseases in rice and wheat, including blast, stripe rust, black-streaked dwarf virus, and sheath blight. This approach effectively mitigated confounding effects associated with population structure in predictive modeling. Furthermore, feature selection was optimized by incorporating SNPs identified through genome-wide association studies (GWAS), achieving an optimal balance between marker density and computational efficiency (Liu et al., 2024). These advances highlight the potential of integrating genomic data with machine learning algorithms to accelerate the identification and utilization of disease-resistant germplasm in rice breeding.

### 5.2 AI-assisted PS for enhanced disease resistance

Accurate PS are critical steps in the development of disease-resistant varieties. These processes support selection decisions by quantifying resistance levels, guiding which lines are retained as parental materials or advanced in the breeding pipeline. Traditionally, phenotypic selection relied on breeders to visually assess and empirically evaluate plants based on simple metrics, such as lesion area or disease incidence (González-Camacho et al., 2018). Although these methods are cost-effective and require minimal equipment, they are inherently low-throughput, labor-intensive, and subjective. The phenotypic characteristics of plant diseases can be represented by diverse data types, including counts, multivariate counts, and images. Deep Learning (DL) has demonstrated outstanding performance in characterizing and predicting these phenotypic data for disease resistance in crops. To address the diverse nature of plant disease damage, researchers have developed various DL algorithms. These include Bayesian regularized neural networks—which are capable of genome-based prediction using Bayesian regression for ordinal data—and Poisson-based deep neural networks (Montesinos-Lopez et al., 2021). While portable spectrometers, UAVs, and satellite remote sensing enable non-destructive measurement of disease-resistance traits, the predictive accuracy of these data varies markedly. Linear regression models with marker matrices achieve high accuracy (Zhu et al., 2021), in contrast to the lower performance of NIRS-based phenomic methods. This progression signifies a broader transformation in plant phenomics, driven by the integration of IoT and AI for high-throughput trait quantification in precision breeding (DeSalvio et al., 2022; Jackson et al., 2023).

A deep learning-based video detection system was proposed for the identification and diagnosis of plant diseases and pests. This system enables rapid recognition by decomposing video sequences into frames analyzed via Faster R-CNN and YOLO v3 for real-time processing (Li et al., 2020). The methodology involves decomposing video sequences into individual frames, which are then analyzed using the Faster R-CNN architecture for object detection. Complementing broad-spectrum monitoring, specialized AI systems have been developed for precise, in-field evaluation of specific diseases. For instance, a multi-task framework introduced for accurate classification and severity analysis of BLB. This approach first generates multiclass segmentation masks of lesions, then employs a dual-path attention mechanism to refine features from both the original RGB image and the segmentation mask. These enhanced features are fused and processed by a lightweight MobileNetV2 classifier, achieving a test accuracy of 96.23% for severity prediction and demonstrating strong robustness in diverse environmental conditions, confirming its real-world deployment potential. Its design supports deployment of edge devices, facilitating real-time, in-field disease management (K. M et al., 2025). In parallel, a hyperspectral imaging-based approach was developed for in-field detection of plant diseases, integrating a line-scan hyperspectral camera with a convolutional neural network (CNN) trained directly on field-acquired spectral data (Almoujahed et al., 2025). These advancements highlight the potential of combining deep learning and hyperspectral imaging for enhancing the accuracy and efficiency of plant disease in agricultural practices.

## 6 Future perspectives

Several key questions and challenges persist. For instance, how can we effectively counteract the rapid evolution of Xoo, especially its capacity to subvert the rice immune system via effector proteins? The downstream signaling components through which R proteins activate plant defense responses remain largely unclear. Moreover, given that most disease resistance genes adversely affect rice yield, resolving the trade-off between disease resistance and agricultural productivity remains a critical challenge. How can novel disease resistance genes be effectively identified and engineered? Furthermore, how can insights from characterized resistance genes guide this discovery and optimization process? 

QTL-mediated resistance is typically non-race-specific and highly durable. To date, more than 100 quantitative trait loci (QTLs) conferring resistance to BLB have been mapped in cultivated and wild rice accessions, as well as in induced and natural mutant populations (Yang et al., 2021). The polygenic nature of BLB resistance involved numerous QTLs with individually small effect. Most QTLs for BLB resistance are mapped to a region of 10–30 cM, due to limited marker density and low recombination frequency in mapping population. It is difficult to accumulate multiple QTLs with small effects in breeding. Challenges are now being overcome through high-resolution sequencing and AI-driven genomic selection.

AI has shown considerable promise in disease-resistant plant breeding, its full potential remains constrained by persistent challenges. These challenges can be categorized into technical and practical barriers: the former includes the lack of large-scale, standardized datasets and the need for more robust algorithms, while the latter involves high computational demands and a significant learning curve for breeders. Together, these issues pose formidable barriers to the widespread adoption and continued development of AI-powered breeding strategies for creating new disease-resistant cultivars.
